# Bison and bighorns: Assessing the potential impacts of reintroducing a large herbivore to a mountainous landscape

**DOI:** 10.1002/ece3.11008

**Published:** 2024-02-26

**Authors:** Peter J. Whyte, Darcy C. Henderson, Karsten Heuer, Adam T. Ford

**Affiliations:** ^1^ Department of Biology University of British Columbia—Okanagan Kelowna British Columbia Canada; ^2^ Parks Canada Banff National Park Banff Alberta Canada; ^3^ Canadian Wildlife Service Environment & Climate Change Canada Kelowna British Columbia Canada

**Keywords:** bighorn sheep, bison, competition, facilitation, habitat selection, *Ovis canadensis*, reintroduction, resource selection

## Abstract

The reintroduction of wildlife can have significant ecological impacts by altering the flow of energy in food webs. Recently, plains bison were reintroduced to part of Banff National Park after a 150‐year absence. The large herbivore's reintroduction was expected to have far‐reaching effects on the ecosystem due to its significant energy requirements and interactions with habitat and other sympatric species. This study explores the impacts of bison reintroduction on the movement and resource use of another large‐bodied grazer, the Rocky Mountain bighorn sheep. Between 2018 and 2021, we collected data from GPS collars fit on 39 bighorn sheep and 11 bison. We analyzed home range patterns, resource selection, and interactions to investigate the potential for interspecific competition, facilitation, and resource complementarity. At the population level, bison and bighorn sheep exhibited low levels of spatial overlap and there was strong evidence of resource separation in all seasons. Interactions between species did not appear to affect sheep movement rates; however, we did see differences in forage selection patterns for sheep with overlapping home ranges with bison. Collectively, results did not support the potential for competition or facilitation between bison and bighorn sheep and instead provided the strongest evidence of complementarity.

## Graphical abstract

Reintroducing North America's largest land mammal can have significant ecological impacts, particularly on the species they interact with. Using GPS data, we analyzed space and resource use of a recently reintroduced herd of plains bison to Banff National Park, to investigate potential interactions with another co‐occurring grazing ungulate, the Rocky Mountain bighorn sheep. Comparing the potential for interspecific mechanisms, our study provides further evidence, that where suitable habitat exists, co‐existence between lost species assemblages may be achieved meeting social and ecological goals.

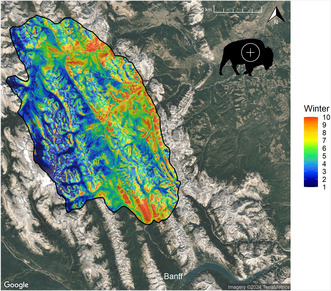

## 1 INTRODUCTION

Species reintroductions are a cornerstone of wildlife restoration efforts worldwide (IUCN, 2013). By re‐establishing populations, reintroductions can bridge fragmented populations (DeCesare & Pletscher, 2006; Seddon, 2010), enhance genetic diversity (Barbosa et al., 2021), restore predator–prey dynamics (Ford, 2021; Fortin et al., 2005; Mao et al., 2005) and reverse species extirpations (Spalton et al., 1999). Wildlife reintroductions can also alter food web dynamics as trophic relationships are renewed or changed.

Ecological relationships between introduced and species already present in the system may affect the success of the reintroduction efforts. For species in the same trophic level, such relationships may be competitive (i.e., shared use of limited resources) or facilitative (Arsenault & Owen, 2002; Stachowicz, 2001). Competition can be indirect through exploitative competition, where one species reduces shared resources limiting the availability to another (Hopcraft et al., 2014; Pianka, 1981; Tilman, 1987). Competition can also be direct through interference competition, where one species reduces access to resources to another species through physical displacement or aggressive behavior (Ferretti & Mori, 2020; Putman, 1995). Facilitation implies that one species is enhancing access to resources for another species (Stachowicz, 2001). A third type of interaction is resource complementarity, in which species partition resources such that there is limited potential for competitive or facilitative interactions (Hoopert, 1998; Vanelslander et al., 2009).

Measuring interspecific competition and facilitation can be challenging in systems dominated by large, free‐roaming mammals. For example, direct evidence of competition in wild populations would include direct measures of resource abundance, species‐specific rates of resource consumption, and demographic responses such as recruitment or survival (Sinclair, 1985; Tilman, 1987). Such evidence is more accessible in mesocosm experiments where species presence and food availability can be manipulated by researchers (Thompson Hobbs et al., 1996).

An indirect approach to testing if species reintroductions are leading to competition or facilitation among large mammals is to examine overlap in space and resources (Lowrey et al., 2018; Schmidt et al., 2000). Under this approach, resource competition may occur when species use the same resources in the same location; interference competition occurs when species use the same resources but in different locations. The effects of presence or proximity between species add further evidence to distinguish these interactions. If proximity between individuals from two species weakens resource selection or causes displacement (Ferretti et al., 2011; Stewart et al., 2010), it suggests that one species is interfering with the other. For example, interactions between roe (*Capreolus capreolus*) and fallow deer (*Dama dama*) can result in roe deer commonly retreating or avoiding feeding sites used by the latter (Ferretti et al., 2011). If proximity strengthens resource selection, it suggests that one species is facilitating resource access for the other. If species do not overlap in space or resource use, it suggests that they are partitioning resources. Testing hypotheses about species interactions using this indirect approach is possible with a combination of GPS telemetry and resource selection analyses. Further, and because this approach to measuring interactions is indirect, we shall hereafter refer to it as testing for the potential of competition, facilitation, or complementarity.

In 2018, plains bison (*Bison bison bison*) were reintroduced to Banff National Park (BNP) Canada, where the species has been absent for over 150 years. Bison are habitat generalists and bulk grazers that occupy a wide variety of climates, topographies, and vegetation types. Preliminary studies suggest that BNP's reintroduction zone supports ample habitat for bison (Steenweg et al., 2016); however, the area is situated at bison's historical western distributional edge (Farr & White, 2022). Unlike the sweeping grasslands for which this species is best known to occupy, BNP is characterized by narrow mountainous valleys, discontinuous grazing habitats, lower forage quality, and deeper snowpacks. These biophysical conditions force bison into valley bottoms during the winter where they might interact with other ungulates, like Rocky Mountain bighorn sheep (*Ovis canadensis*; hereafter, sheep). The grazing activity of bison has been observed to affect the emergence of newly growing plants during the spring green‐up period (Geremia et al., 2019) and in certain environments contribute to increased diversity in vegetation (Ratajczak et al., 2022). Because sheep are considered specialized grazers, the effects of bison on forage could support sheep's access to resources. For example, bison are known to ‘engineer the green wave’ by reducing coarse, rank grasses and leaving behind more nutritiously dense food (Geremia et al., 2019). Species like sheep may therefore potentially benefit from the grazing of bison. On the other hand, bison are a large gregarious species and can reduce forage biomass such that it limits their own population (Fuller et al., 2007) and presumably that of other species.

After more than 150 years since bison and sheep co‐habituated this landscape, this study aimed to delineate the spatial ecology of sheep residing around the reintroduction zone and assess the likelihood of new ecological interactions with bison. Using a combination of GPS telemetry on bison and sheep, as well as satellite‐derived measures of resources (e.g., foraging habitat), we tested three mutually exclusive hypotheses to explain bison‐sheep interactions. The Competition Hypothesis makes several predictions regarding the movement and resource use by bison and sheep. First, we predicted that there will be low home range overlap between sheep and bison, but high resource overlap. Low home range overlap occurs because at least one of the species is avoiding the other. We also predicted that bison and sheep interactions would cause sheep movement rates and their distance to bison to increase relative to before an interaction. Finally, if bison reduce the amount of forage available for sheep, then sheep living near bison will increase their use of foraging habitats as it becomes more available (i.e., a Type I functional response; Dupke et al., 2020; Holling, 1957), whereas in the absence of bison, sheep's use of foraging habitat will be asymptotic as more foraging habitat is available (i.e., a Type II functional response).

We contrast the Competition Hypothesis with the Facilitation Hypothesis, in which we predicted sheep would (a) have high spatial and resource overlap with bison; (b) increase their use of foraging habitat in the presence of bison; (c) show preferential selection for areas that bison used; and (d) following interactions with bison, sheep do not increase movement speeds or distance from bison. Finally, we tested the Complementarity Hypothesis where neither high spatial nor resource overlap is present, and bison has minimal impact on sheep habitat selection or movements.

## 2 MATERIALS AND METHODS

### 2.1 Study area

**FIGURE 1:**
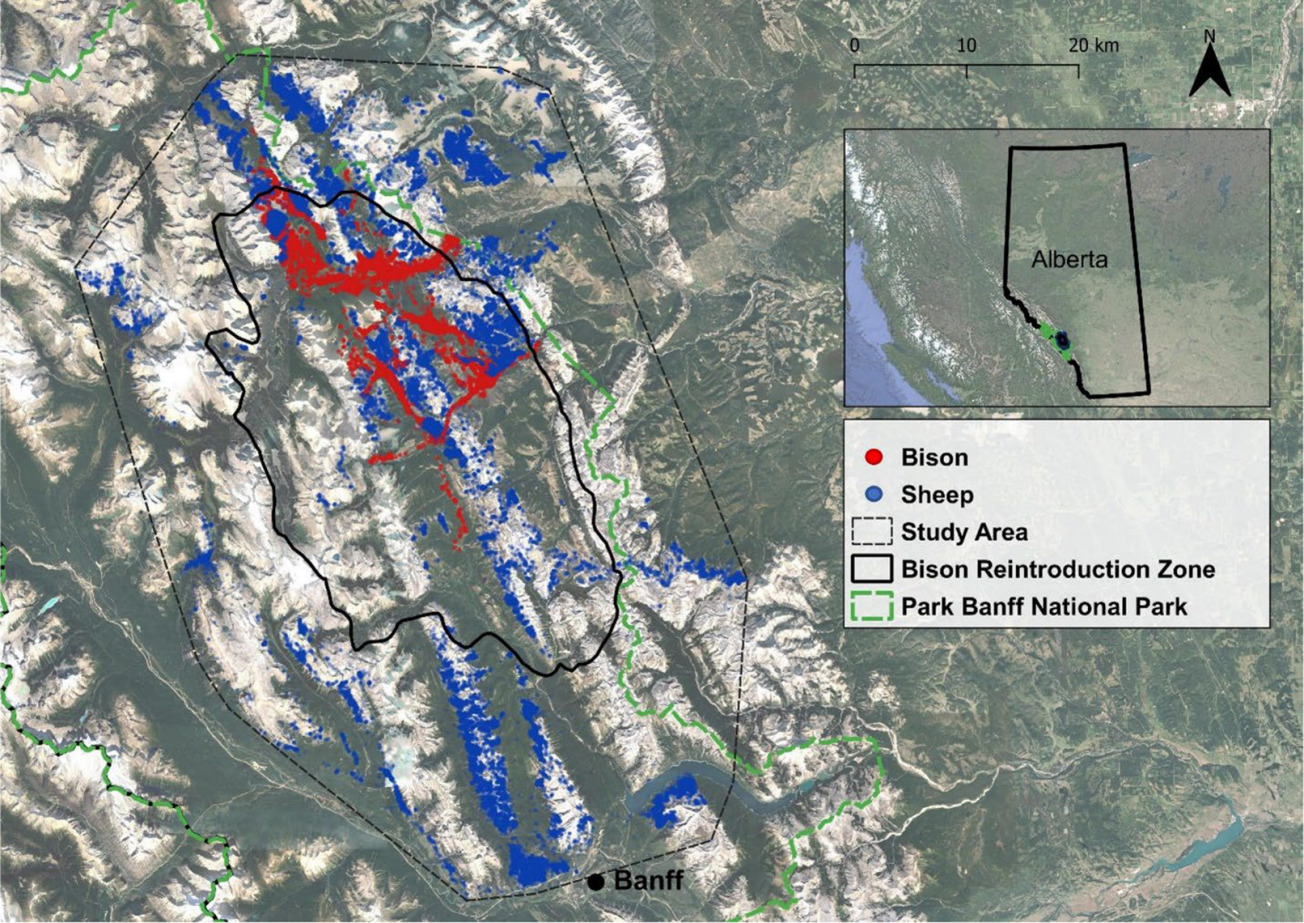
GPS fixes for 11 collared bison (August 1, 2018–September 09, 2021) and 39 sheep (October 10, 2019–September 09, 2021) located along Banff National Park's eastern boundary. The study area (black dashed polygon) encapsulates the bison reintroduction zone and was defined by collared sheep and bison movements using a minimum convex polygon.

The study area was defined by the movements of collared bison and sheep and is located within, and adjacent to, the northern reaches of BNP in the Canadian Rocky Mountains (Figure 1). These movements were based on data collected from GPS telemetry of 11 bison (August 1, 2018–September 09, 2021) and 39 sheep (October 10, 2019–September 09, 2021). Bison are gregarious, such that the collared animals who represent >10% of the reintroduced bison population, are representative of herd‐level movements.

The study area is characterized by steep, rocky, colluvial terrain to the west (elevation: 1400–3600 m) with large valleys extending eastward to the Rocky Mountain foothills. In this region, a continental climate regime dominates resulting in long cold winters, cool summers, and relatively sparse precipitation (Strong & Leggat, 1992). The area is thought to contain some of the best bison habitat in the park (Steenweg et al., 2016), and some of the region's best sheep winter range (Skjonsberg, 1987). While sheep moved through the area freely, bison are contained to a target reintroduction zone that is bounded by natural high ridges, strategically placed drift fencing in valley bottoms (Laskin et al., 2020), and reactive hazing/herding responses from Parks Canada personnel (Zier‐Vogel & Heuer, 2022).

### 2.2 Data collection

Starting in 2018, 15 sheep were fitted with GPS collars inside the bison reintroduction zone and another 24 in the greater BNP (October 10, 2019–September 09, 2021, *n* = 39; fixes = 196,103; 2‐h fix rate); collared sheep consisted of both male (*n* = 15) and females (*n* = 26). Using aerial net gunning or ground darting techniques captures targeted sheep from known winter ranges. Upon capture, sheep were fitted with a GPS collar (Survey Iridium, VECTRONIC Aerospace GmbH, Berlin, Germany) and marked with a numbered ear tag. Using similar techniques, 11 female bison were collared before and since being released in 2018 (August 1, 2018–September 09, 2021; *n* = 11; fixes = 53,965; 2‐h fix rate); these collared individuals represented general bison movements as the whole herd remained in one or two relatively cohesive groups (Zier‐Vogel & Heuer, 2022). We censored imprecise locations by removing fixes that were non‐3D and associated with abnormally high step speeds >20 km/h and/or above the 99 percentiles for each species.

## 3 DATA ANALYSES

### 3.1 Spatial overlap

We examined home range overlap for individual animals and then assessed group overlap from each season. We removed animals that had <75% of a year's accumulated GPS fixes resulting in 33 sheep and 9 bison. To examine annual home range overlap among individuals, a utilization distribution (UD) derived from a Brownian bridge movement model (BBMM) was created for each collared animal at a resolution of 150 m using a 20 m GPS error parameter. Once UDs were created, annual home ranges were defined as the area within 95% of the probability distribution. We also examined home range overlap at a group scale for each season. For group UDs, we first created seasonal UDs using the same method for all individuals and then averaged UDs by season for each respective species. We included all seasonal bison UDs and only female sheep whose annual home ranges were found to overlap with bison (*n* = 5). We used two methods to measure home range overlap. First, we calculated the percent overlap by the proportion of the sheep UDs that were occupied by the bison UD (Fieberg & Kochanny, 2005). Second, we estimated Bhattacharyya's Affinity Coefficient (BA; Bhattacharyya, 1943; Fieberg & Kochanny, 2005). The BA coefficient measures the similarity between two statistical samples (UDs) that are assumed to be independent and produces a value between 0 (no overlap) and 1 (identical distributions).

### 3.2 Sheep habitat selection and overlap

Sheep are sexually dimorphic and habitat selection is likely to vary between rams and ewes (Ruckstuhl, 1998). As females are likely to have a stronger effect on population dynamics, we chose to build habitat models using GPS fixes from only females who utilized habitats within the park (*n* = 24). Based on recent research on sheep habitat selection in the Canadian Rocky Mountains (Poole et al., 2016) and observed bison movements, resource selection was assessed in four seasons: Winter (December 1–April 30); Spring (May 1–June 30); Summer (July 1–August 31); Fall (September 1–November 30). To examine seasonal sheep habitat selection, a collection of 30 m × 30 m raster‐based land cover and terrain attributes were selected based on previous sheep research (DeCesare & Pletscher, 2006; Lowrey et al., 2018; Poole et al., 2016). Land‐cover layers were derived from the Vegetation Resource Inventory (VRI) system (Sandvoss et al., 2005). We pooled fine‐scale vegetation and land cover layers of the VRI into five groups (forest, shrubs, grass and forbs, rocks and rubble, snow, and water).

We derived topographic variables (elevation, slope, and terrain ruggedness) using a digital elevation model (DEM; Gillies et al., 2006). To calculate terrain ruggedness, we used the vector ruggedness measure (VRM) based upon the DEM to calculate the three‐dimensional orientation of grid cells within a neighborhood scale set at 7 × 7 cells (Poole et al., 2016; Sappington et al., 2007). Escape terrain was defined as slopes >75% and the distance to escape terrain was measured in meters (Poole et al., 2016). To represent heat load, we transformed the aspect in degrees to a range of −1 and 1, representing north‐northeast to south‐southwest respectively (Lowrey, 2018). For snow depth, we used the Canadian Hydrological Model (CHM) (Marsh et al., 2020) configured as described in Vionnet et al. (2021) for estimating snow depths in BNP. Atmospheric forcing input to CHM was the 2.5 km Environment and Climate Change High‐Resolution Deterministic Prediction System (HRDPS; Milbrandt et al., 2016). Mean snow depth was calculated from daily estimates for the defined winter season during the years 2020–2021. Continuous variables for elevation and slope were scaled (i.e., subtract mean, divide by standard deviation), to better match the categorical binomial structure and help with model convergence (Zuur et al., 2007).

We tested sheep's selection of areas with high bison utilization using the same utilization distributions created for annual home ranges. Because our goal was first to create predictive habitat maps for sheep, and examine bison use within them, we initially did not include the bison UD variable within the top model; the bison variable was tested in best‐performing seasonal models after predicted sheep habitat maps were created.

To estimate resource selection, Resource Selection Functions (RSFs) were created using logistic regression with a binomial generalized linear mixed‐effects model (GLMM; Boyce et al., 2002). To account for variability between individuals, we incorporated a random intercept into the GLMM for each individual and evaluated resource selection within their home range (i.e., 3rd order selection; Boyce et al., 2002; Johnson, 1980). The addition of a random intercept within the model helps account for unbalanced sampling between individuals and the potential lack of independence between GPS fixes (Zuur & Ieno, 2009). The RSFs were built using a used/available design (Manly et al., 2002), where used (=1) represents individual collar uploads and available (=0) were sampled within each individual's 95% minimum convex polygon (MCP) from the *amt* package (Signer et al., 2020). For each used point, 10 available points were created randomly within the MCP using GIS software. We set weights for used locations to 1 and available to 1000 (Whittington et al., 2022).

Before fitting models, collinearity was evaluated using Pearson's correlation test with the *cor* function (base R; R Core Team, 2020); no predictor variables were shown to have a correlation factor >0.6, and therefore, all were included in the global model (Whittington et al., 2022). All models were created using the *lme4* package (Bates et al., 2015) and AICc was used to determine the top model. Once a top model structure was identified, we used the *dredge* function from the *MuMIn* package (Bartoń, 2022) to identify which predictor variables significantly contributed to model performance; the priority of the model was to determine high‐quality habitat. If the top models were within ∆ <2.0 AICc, we assessed the relative variable importance by comparing Akaike model weights and selected the model with the higher weight of evidence in favor. Models were validated using five‐fold cross‐validation repeated 100 times and an averaged Spearman's rank correlation coefficient (Boyce et al., 2002).

Using GIS, we projected the coefficients from the RSFs to a map and then created 10 equal‐area quantile bins of the resulting map (Morris et al., 2016). To measure resource overlap, we then calculated the frequency of bison GPS fixes across the sheep RSF bins. We also inspected the proportion of used sheep locations found within each bin. Finally, we examined if bison utilization within the reintroduction zone had any effect on sheep habitat selection. To test this, we added a variable of the bison UDs to the above‐mentioned top models to test if model performance increased (i.e., that the AICc was lowered by more than 2 units).

### 3.3 Resource overlap

For this analysis, we focused on testing differences in selection for variables related to the topographic position and forage (elevation, slope, grass cover, and shrub cover). We used the same variables as the RSF for elevation and slope, however, converted the binomial land cover class to a continuous variable by performing neighborhood analyses within 500 m circular buffers. Before testing the strength of selection between species, we plotted the distribution of used GPS fixes and calculated a mean value per season.

To test if resource selection between sheep and bison was significantly different across seasons, we used latent selection difference (LSD) functions (Fischer & Gates, 2005; Latham et al., 2011; Roever et al., 2008). For our purposes, negative coefficient values indicated relatively lower use of a resource by sheep relative to its use by bison; a positive coefficient indicates relatively higher use of a resource by sheep relative to its use by bison. When LSD coefficient estimates are not significant (i.e., the estimate overlaps with 0), resource selection between species is assumed to be not significantly different (i.e., the individuals similarly used or avoided that resource). The LSD analysis allows a direct comparison of used resources between species; however, the lack of a defined availability limits inference to an area where habitats are the same (Viejou et al., 2018). To meet this assumption, we compared female sheep that were found within the core reintroduction zone (*n* = 5) to female bison with a similar number of GPS fixes (*n* = 5). We added a random intercept to account for variability between pairs and random slope to account for varying elevations between individuals.

### 3.4 Interspecific interactions

To maximize the sample size of direct interactions, we took advantage of an additional year's worth of GPS movement data and included male and female sheep (October 10, 2019–November 23, 2022, *n*_sheep_ = 436,470; March 5, 2018–November 23, 2023, *n*_bison_ = 156,001). We assessed if sheep movement speed was affected by proximity to bison in time and space. We followed a methodology assessing simultaneous fixes between two species (Middleton et al., 2013; Miller, 2015) and, using the *wildlifeDI* package (Long et al., 2022), we defined interactions as any simultaneous GPS fixes within half of the fix‐rate (60 min; Long et al., 2022) that were within what we considered a conservative spatial window (200 m). These parameters were based upon estimates by Geist (1971) that sheep behavioral reaction (e.g., alarm posturing) to predators and other approaching sheep is short‐lived when animals are farther than 350 m apart. If simultaneous fixes within the spatial window occurred for longer than 60 min, we grouped fixes into contact phases and assessed each interaction by the first fix within a phase (Long et al., 2022).

Using a generalized additive model, we used a bivariate smoother on time to contact and distance to bison to test for the effects of an interaction on sheep movement speed (m/h). Movement speed was calculated by dividing step length by the time between sequential fixes. We expect that if sheep ‘flee’ from bison, then there will be an increase in movement speed as proximity to bison decreases. We log‐transformed the movement speed variable to satisfy assumptions of normality and included a first‐order autoregressive term to account for the spatial and temporal autocorrelation introduced by tracking data.

### 3.5 Sheep forage resource selection

Grass, forbs, and shrub cover are associated with the types of forage used by both sheep and bison (Geist, 1971; Stelfox, 1976). We assessed whether the availability of grass, forbs, and shrub cover affects sheep's use of foraging habitat in the presence and absence of bison. If resources are not limited, we expect that sheeps' use of these resources will asymptote as its availability increases (i.e., A Type II or Type III functional response). Conversely, if these resources are limiting, we expect sheeps' use of these resources to increase as its availability increases (i.e., a Type I functional response). We used General Additive Mixed Models (GAMMs) to compare the use of foraging habitat by two groups of sheep: (1) those with home ranges that overlapped with bison (*n* = 9) and (2) those whose home ranges did not overlap with bison (*n* = 24). For the response variable, we used the counts of individuals' GPS fixes found within the grass, forbs, and shrub land cover types. To define resource availability, we used GIS software to randomly sample land cover within the individual's home range using a 1:1 ratio of observed and available locations (see RSF methods). Using the *mgcv* package (Wood, 2017), we ran GAMM models with a Poisson distribution and log link function (Zuur et al., 2007).

To incorporate uneven sample sizes, we offset the count data for the forage type by the total number of GPS fixes and then allowed for a random intercept for each animal. We then estimated selection curves for each group of sheep (i.e., Overlap with bison or No Overlap with bison). Overlap was defined as male and female sheep who exhibited a >1.0% mean spatial overlap with bison (*n* = 9); refer to methods for Spatial Use and Overlap Between Bison and Sheep. Smoothing terms were based on thin plate regression spline, and then, we used the default generalized cross‐validation approach found within the *mgcv* package. We determined the size or scale for each spline (*k*) using the gam. check() function, which runs a simulation‐based check to help identify when splines are too small by providing an associated *p*‐value (Wood, 2017). Following the methodology for other GLMs, we verified these models visually using validation plots (Zuur et al., 2007).

## 4 RESULTS

### 4.1 Spatial overlap

We created annual UDs for 33 out of the 39 sheep (female = 21, male = 12) and 9 bison (female = 9; Table 1; Table S1). Some collared animals did not accumulate enough GPS fixes to create annual UDs. Sheep 95% UDs size averaged 22.42 ± 9.78 km^2^ and bison were 38.22 ± 5.41 km^2^ (±standard deviation). Nine sheep exhibited a mean annual spatial overlap of >1% with bison and the highest mean spatial overlap was 21% (Table 1). Mean Bhattacharyya's Affinity (BA) coefficient for individual sheep was relatively low and ranged from 0 to 0.08. For group UDs, the level of spatial overlap between species varied slightly by season, but overall, was relatively low. Fall had the highest level of overlap at 4.73% and winter was slightly lower at 3.41% (Table 2). BA coefficients were also relatively low between‐group UDs. Again, the overlap was greatest in winter and fall at 0.04.

**TABLE 1:** Utilization distribution (UD) size and mean overlap (±SD) of sheep who overlapped (*n* = 9) or interacted (*n* = 11) with bison.

Sex/sheep ID	UD size (km^2^)	Mean overlap (%)	Mean BA	# Interactions
Female 47	16.74	1.00 ± 1.00	0.00 ± 0.00	–
Female 39	34.85	1.00 ± 0.00	0.01 ± 0.00	–
Female 43	26.89	1.00 ± 0.00	0.01 ± 0.00	2
Female 45	16.13	2.00 ± 1.00	0.00 ± 0.00	1
Male 38	35.84	2.00 ± 1.00	0.01 ± 0.00	2
Female 48	31.34	3.00 ± 0.00	0.06 ± 0.01	–
Male 50	33.64	4.00 ± 1.00	0.05 ± 0.01	8
Male 49	36.92	4.00 ± 1.00	0.08 ± 0.01	10
Male 46	16.00	13.00 ± 4.00	0.04 ± 0.01	1
Male 31	10.24	21.00 ± 6.00	0.05 ± 0.02	–
Female 44	N/A	N/A	N/A	1
Male 72	N/A	N/A	N/A	2

*Note*: Overlap was measured by the percentage area overlapping in the sheep UD and the Bhattacharyya's affinity coefficient (BA).

**TABLE 2:** Pooled utilization distribution sizes (km^2^) for female sheep (*n* = 5) who overlapped with collared bison (*n* = 9) for each season.

Season	Bison (km^2^)	Sheep (km^2^)	Overlap (%)	BA
Winter	21.76	25.41	3.41	0.04
Spring	20.60	42.20	0.00	0.00
Summer	51.90	97.38	2.85	0.01
Fall	30.75	32.39	4.73	0.04

*Note*: Overlap was measured by the percentage area of overlap in the sheep UD and Bhattacharyya's affinity coefficient (BA).

### 4.2 Sheep habitat selection and overlap

All seasonal models were constructed using GPS fixes of 24 female sheep and the same habitat variables, except winter which included snow depth, and summer when NDVI was included. The final seasonal models were shown to have excellent predictive accuracy (>0.99; Table S2). Alternatively, the randomly sampled available data exhibited extremely poor predictive capabilities.

**FIGURE 2:**
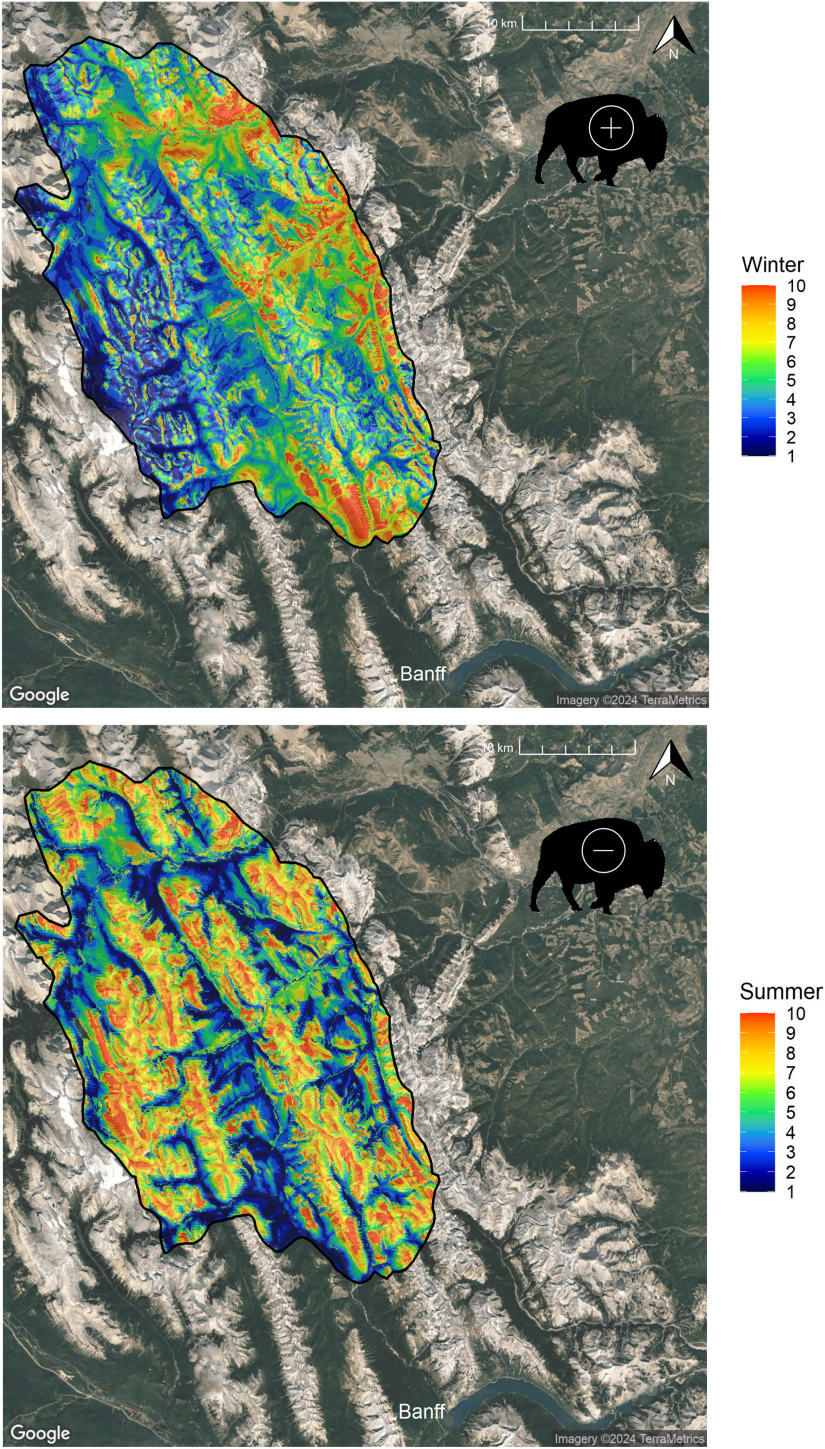
Predictive maps derived from resource selection functions for sheep overlayed on the bison reintroduction zone within Banff National Park. The relative probability of sheep selection was divided into 10 bins where red (10) depicts a higher probability of selection, and blue low (1). The bison icon indicates whether adding a bison variable to the resource selection models resulted in a positive or negative coefficient (i.e., sheep selected for areas where bison used most, or avoided).

**FIGURE 3:**
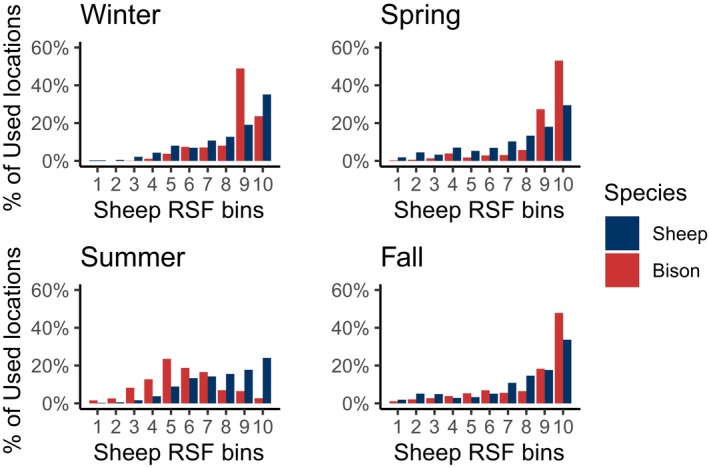
The proportion of bison (*n* = 9) and sheep (*n* = 24) GPS fixes found within seasonal sheep equal‐area RSF bins. Higher bin values (10) are indicative of areas with a higher relative probability of selection by sheep.

Across all seasons, sheep resource selection was characterized by a preference for mid‐elevation terrain, warmer aspects, and more rugged steeper slopes within proximity to escape terrain (Figure 2; Table S4). Sheep avoided areas closer to permanent water bodies and areas with higher mean snow depths in winter. For every season except summer, sheep were selected for grass and shrub cover types. Overall, rugged terrain had the strongest effect on sheep selection. Within the reintroduction zone, there were high proportions of sheep locations found within the high bins (9–10) of the projected sheep RSF – further validating the RSFs (Figure 3).

There were relatively high proportions of bison fixes (>40%) found in the high‐value bins of the projected sheep RSF in the spring, fall, and winter (Figure 3). In summer, the proportion of bison fixes were spread out across a wider range of lower‐value RSF bins.

Bison utilization was shown to improve model predictability for the RSF models during each season (Table S3). During winter and fall, we found that bison utilization increased the relative probability of sheep use (Table 2). Conversely, during spring and summer, higher bison utilization decreased the relative probability of sheep use. For each respective seasonal model, the addition of a bison variable did not change the relationship of any of the other variables.

### 4.3 Resource overlap

The latent selection difference (LSD) analysis provided strong evidence suggesting separation in resource selection for all biologically important variables (Table 3). For our LSD analysis, a positive coefficient indicates stronger selection for sheep and a negative coefficient indicates stronger selection for bison. For all seasons, sheep were strongly selected for higher elevations and steeper slopes relative to bison (Table 3). Bison selected for land cover types with higher proportions of grass and forbs, while sheep showed a stronger selection for higher proportions of shrubs for every season except summer.

**TABLE 3:** Coefficient estimates and 95% confidence intervals for latent selection differences (LSD) models comparing the relative habitat selection between bison (coded as 0) and sheep (coded as 1) for four biologically important variables (elevation, slope, grass and forbs, and shrubs).

Variables	Winter		Spring		Summer		Fall	
	Estimates	*p*	Estimates	*p*	Estimates	*p*	Estimates	*p*
Elevation	5.14 (4.83 to 5.46)	<.001	4.65 (4.24 to 5.07)	<.001	4.38 (4.10–4.66)	<.001	3.06 (2.82 to 3.30)	<.001
Slope	3.33 (3.09 to 3.57)	<.001	2.23 (1.98 to 2.47)	<.001	1.80 (1.67–1.93)	<.001	2.79 (2.59 to 2.98)	<.001
Grass/forbs	−5.54 (−7.10 to −3.99)	<.001	−3.34 (−4.50 to −2.18)	<.001	−19.94 (−21.26 to −18.61)	<.001	−4.77 (−5.75 to −3.79)	<.001
Shrubs	9.50 (8.56 to 10.43)	<.001	3.68 (2.75 to 4.62)	<.001	−7.38 (−7.88 to −6.87)	<.001	1.90 (1.17 to 2.63)	<.001
Observations	25,608		10,566		13,665		9159	
Marginal *R* ^2^/conditional *R* ^2^	.953/.966		.924/.947		.853/.949		.889/.917	

*Note*: A positive coefficient indicates stronger selection for sheep and a negative coefficient indicates stronger selection for bison.

### 4.4 Interspecific interactions

**FIGURE 4:**
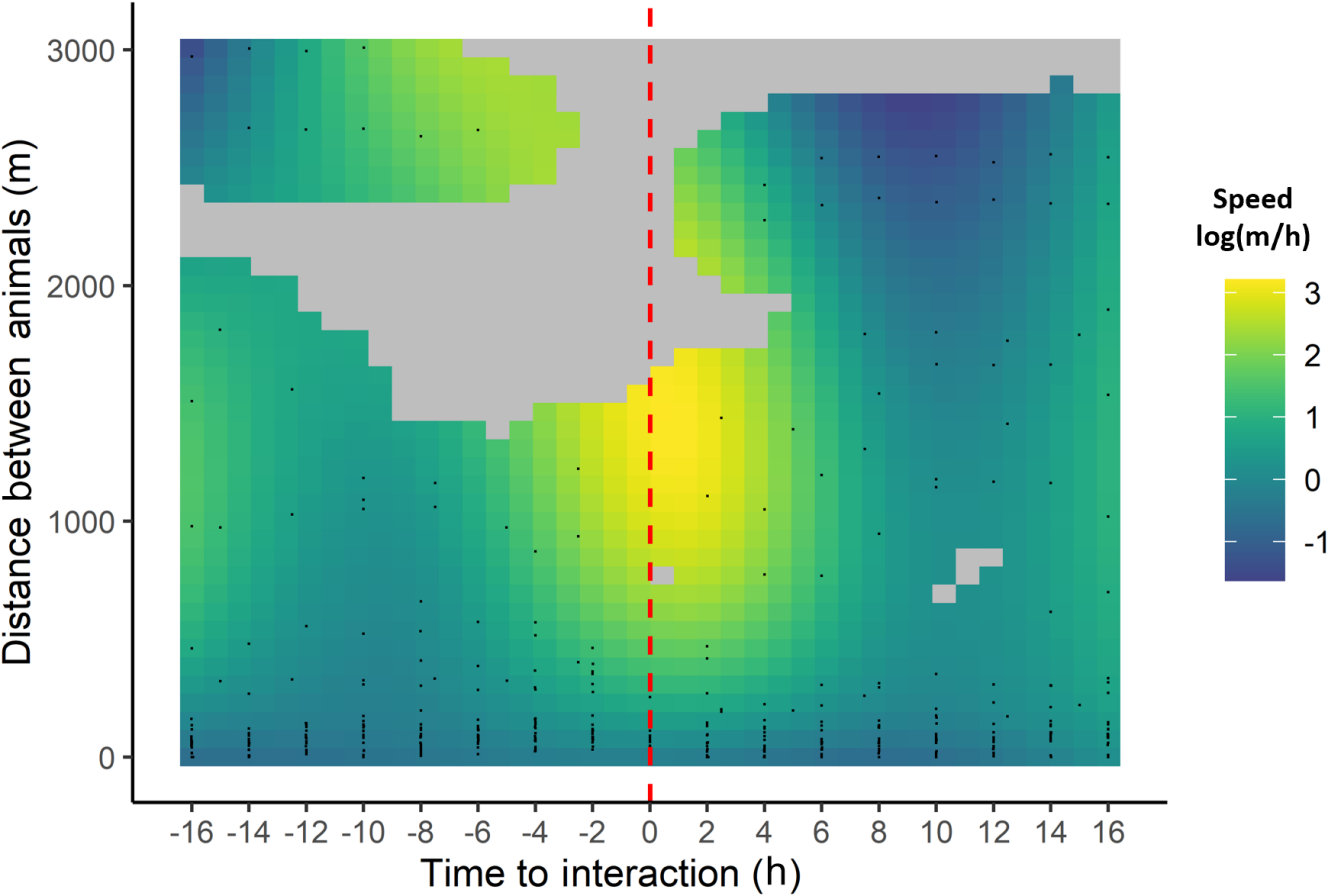
Surface plots showing the predicted effect of distance to bison and time‐to‐contact on sheep movement speed (m/h). We modeled movement rate using 29 interactions (red dashed line) between bison (female) and sheep (male and female) in Banff National Park. Bright colors indicate more rapid movements by sheep.

Between October 19, 2019, and November 23, 2022, there were 29 recorded interactions between individual bison and sheep (male and female). Six interactions lasted for only one simultaneous GPS fix and the mean duration of interactions was 11 h (Table S5). Movement rates of sheep varied as a function of time to interaction and distance to bison (E.D.F. = 13.65, *p* < .001; Table S6). We observed increasing movements by sheep as the distance between bison and sheep decreased (Figure 4). There was an increase in sheep movement speed both before and after an interaction and the effect decreases over time.

### 4.5 Sheep forage resource selection

**FIGURE 5:**
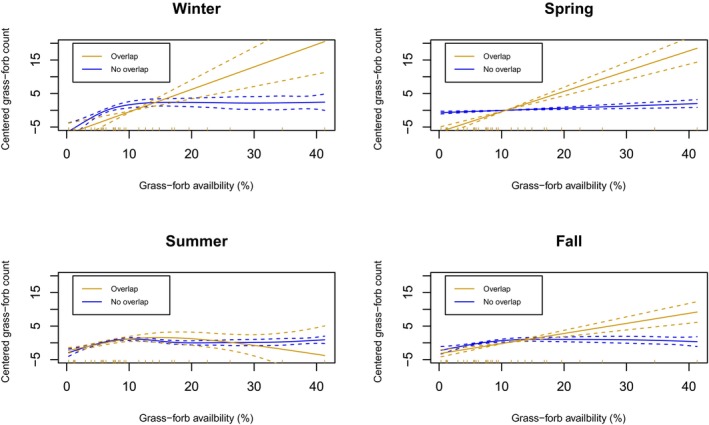
Sheep's selection of the grass forbs land cover type in Banff National Parks' as a function of availability and separated by the status of home range overlap with bison (Overlap *n* = 9; No overlap *n* = 24). Smoothed curves (solid with 95% CI dashed lines) were created using generalized additive mixed models (GAMM) using the count of GPS fixes within grass forbs offset by the total amount of GPS fixes for each sheep (mean‐centered). Vertical dashes along the *x*‐axis represent the distribution of data; due to the paucity of data, curves >25% availability should be interpreted with caution.

**FIGURE 6:**
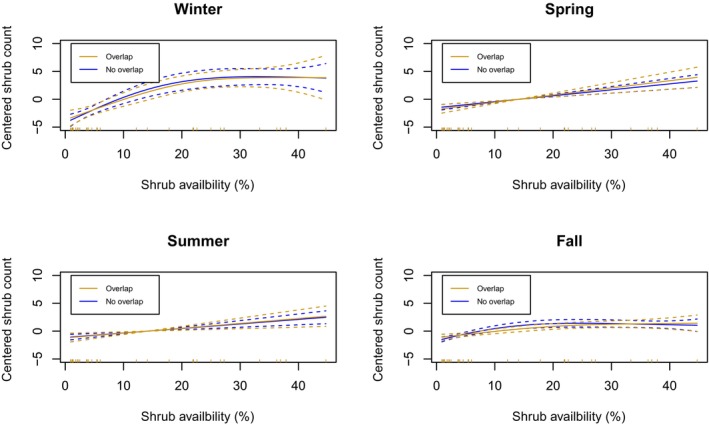
Sheep's selection of the shrub land cover type in Banff National Parks' as a function of availability and separated by the status of home range overlap with bison (Overlap *n* = 9; No overlap *n* = 24). Smoothed curves (solid with 95% CI dashed lines) were created using generalized additive mixed models (GAMM) using the count of GPS fixes within shrub offset by the total amount of GPS fixes for each sheep (mean‐centered). Vertical dashes along the *x*‐axis represent the distribution of data; due to the paucity of data, curves >25% availability should be interpreted with caution.

Sheep that did not overlap with bison demonstrated a slightly positive relationship between the use of grass and forbs and availability for most seasons (Figure 5), except in winter. During the winter, the use of grass and forbs cover increased until ~10% availability and then leveled off (i.e., a Type II functional response). For sheep whose home ranges overlapped with bison, a more constant, positive linear effect for three out of four seasons was evident (i.e., a Type I functional response). This positive relationship appeared strongest in winter and spring. Overall, few sheep experienced a home range with >25% availability of grass and forbs, and therefore model estimates past this point should be interpreted with caution. For shrub cover, sheep use was similar for animals that did and did not overlap with bison (Figure 6). In winter, sheep use of shrub habitat flattened off as the availability within a home range approached 20%. All other seasons demonstrated a weaker positive association which followed a more linear trend.

## 5 DISCUSSION

The potential for competition was not a strong feature of sheep‐bison spatial and resource ecology in BNP. For the potential for competition to occur, we predicted sheep and bison would show patterns of spatial avoidance while selecting similar resources, particularly in winter. In contrast, we observed: (1) low spatial overlap between sheep and bison in all seasons; (2) seasonally‐mediated resource partitioning; (3) sheep selection for areas of higher bison use; (4) no discernable avoidance behavior of sheep to bison; (5) a saturating (Type II) functional response of forage site selection by sheep in the absence of bison suggesting sheep are acquiring adequate forage and a non‐saturating (Type I) functional response in the presence of bison.

The degree of home range overlap between bison and sheep varied slightly by season, but at the population scale, was relatively low. On the individual scale, some sheep shared as much as 20% of their annual home range with bison, but the intensity of use in shared areas remained low (BA values were low). Our results align with Jung, Hegel, et al. (2015), who assessed late winter spatial relationships between a community of ungulates following a bison reintroduction to the Yukon. While low spatial overlap was predicted by the Competition Hypothesis, the use of resources differed among sheep and bison – weakening support of competition. Sheep tended to use different resources than bison in all seasons, particularly in fall, winter, and spring when resources are most limiting. These patterns of resource selection by sheep are consistent with findings elsewhere (DeCesare & Pletscher, 2006; Donovan et al., 2021; Poole et al., 2016).

Areas of higher bison utilization were selected by sheep in the winter; however, it is difficult to determine if sheep had similar relationships in these areas before the reintroduction. Observations of sheep from several decades ago indicate that some of the overlapping areas were used by sheep in the winter (Skjonsberg, 1987). These results suggest bison do not competitively exclude sheep in winter. Alternately, sheep are known to exhibit multiple winter foraging strategies (Geist, 1971), including selecting lower‐elevation grasslands or slopes that are free from snow (Poole et al., 2016; Tilton & Willard, 1982). Bison also select for low snow depths, and thus some of the overlap may be driven by a mutual preference by both species for these types of habitats. Direct observation is needed to confirm whether winter bison trails and cratering behavior provide easier access to grass and forbs for sheep.

When we projected the sheep RSF across the bison reintroduction zone, we found a relatively high proportion of bison GPS fixes within higher‐valued sheep habitats. This implies that if bison or sheep populations increase, further resource and spatial overlap could develop in the future. However, the extent of bison use of areas highly used by sheep was restricted and occurred mostly on two, low‐elevation meadows where observations of sheep are rare. These unique areas were relatively far from sheep escape terrain, so we do not expect these sites to be used by sheep even if bison were not reintroduced.

We did not see a clear change in sheep movement rates following an interaction with bison. For an interaction to occur, displacement (distance between individuals) inherently decreases as the time to the interaction decreases. Although sheep moved faster when close to bison, the effect was consistent both before and after an interaction, suggesting that the increased speed was not solely due to the presence of bison. Additionally, our analysis did not account for slope, which depending on the topography where the interactions took place could have impacted sheep's response. Few studies have demonstrated behavioral changes through interspecific interactions for species that have co‐evolved, particularly in temperate systems (Ferretti & Mori, 2020). Stewart et al. (2002) reported avoidance behavior between mule deer and elk within small temporal windows (<6 h), however, spatial differences were not maintained. Berger (1985), who directly observed a guild of species, including sheep and pronghorn (*Antilocapra americana*), concluded interactions between native species did not suggest dominance of one of the other. Similarly, during interactions with non‐native species (e.g., cattle), sheep increased vigilance, however, no clear avoidance behavior or displacement has been reported (Brown et al., 2010).

We found a Type II functional response – or decelerating intake rate – of land cover associated with forage by (1) sheep in the absence of bison in all seasons and (2) in the presence of bison in the summer. A Type II response is common for many grazers, with forage quality and abundance constraining intake (Bjørneraas et al., 2010; Dupke et al., 2020; Wilmshurst et al., 1999, 2000). If habitat use is related to intake rate, then we may expect similar patterns of saturation when resources are abundant (Dupke et al., 2020; Wilmshurst et al., 1999).

We found a Type I functional response – or linear increase – of forage site use by sheep in the presence of bison during fall, winter, and spring. Because both bison and sheep are predominantly grazers (Knapp et al., 1999; Stelfox, 1976) and previous studies have reported a high degree of diet overlap (Jung, Stotyn, & Czetwertynski, 2015), we expected that bison activity could affect the functional response of sheep use with increasing forage availability. Though the linear response to forage availability could be suggestive of either facilitation or competition, the low spatial extent to which these species overlap at the population level makes it difficult to estimate any impact on sheep fitness caused by bison. Thus, the Type I functional response we observed by sheep in the presence of bison may suggest that bison are limiting access to forage for sheep in spring, winter, and fall. At the same time, there may be confounding variables underlying this finding which limits our confidence. For instance, grassland habitats are less common in the low‐elevation areas where bison occur, when compared to other parts of the landscape. Importantly, this study was limited to inferring these results from the population level and a relatively small number of sheep whose range overlapped with bison. Similar to the results from the interaction analysis, it is important to remember sheep are sexually dimorphic (Ruckstuhl, 1998), which could cause different functional or movement responses for rams or ewes when interacting with bison. To better understand the direct interactions between sheep, bison, and forage across different areas, further research is necessary.

Population densities play a large role in how bison impact vegetation (Larson, 1940) and consequently how they interact with other co‐occurring species (Foca & Boyce, 2022). In our study, there was little evidence suggesting competition is a strong mechanism between bison and sheep. With growing bison populations in BNP, that are limited to a defined range and constrained by active management (Laskin et al., 2020), we acknowledge the potential for bison resource use to change as their population increases. For example, in other jurisdictions where bison are restricted to limited ranges, increasing densities and herbivory have the potential to impact the structure, composition, and distribution of woody plant communities (Beschta et al., 2020). Our results suggest that changing bison densities have the potential to alter mechanisms between bison and sheep, however, these impacts would likely remain isolated to the relatively small areas where these species overlap. As parks and protected areas – often in mountainous environments – continue to provide opportunities for free‐ranging bison restoration efforts, further understanding these species’ ecological impacts remains vital to their long‐term management and social acceptance.

## AUTHOR CONTRIBUTIONS

**Peter J. Whyte:** Conceptualization (lead); data curation (lead); formal analysis (lead); methodology (lead); writing – original draft (lead); writing – review and editing (lead). **Darcy C. Henderson:** Conceptualization (supporting); supervision (supporting); writing – original draft (supporting); writing – review and editing (supporting). **Karsten Heuer:** Data curation (supporting); project administration (supporting); writing – original draft (supporting); writing – review and editing (supporting). **Adam T. Ford:** Conceptualization (equal); formal analysis (supporting); funding acquisition (supporting); project administration (supporting); supervision (lead); writing – original draft (supporting); writing – review and editing (supporting).

## Supporting information


Tables S1–S6.

## Data Availability

All data and R code has been uploaded to a Dryad repository: DOI: https://doi.org/10.5061/dryad.xd2547dp0.
