# Supplementary material for: Bison and bighorns: Assessing the potential impacts of reintroducing a large herbivore to a mountainous landscape
Source: Ecol Evol. 2024 Feb 26;14(2):e11008. doi: 10.1002/ece3.11008 (PMC10897061; doi:10.1002/ece3.11008)

# Supporting Information

**Table** **S1**: Utilization distribution (UD) size and mean overlap (± SD) of individual sheep who overlapped with bison (n=9). Overlap was measured by the percentage area overlapping in the sheep UD and the Bhattacharyya’s affinity coefficient (BA).

| Sex/Sheep ID | UD Size (km^2^) | Mean Overlap (%) | Mean BA |
| --- | --- | --- | --- |
| Female 47 | 16.74 | 1.00 ±1.00 | 0.00 ±0.00 |
| Female 39 | 34.85 | 1.00 ±0.00 | 0.01 ±0.00 |
| Female 43 | 26.89 | 1.00 ±0.00 | 0.01 ±0.00 |
| Female 45 | 16.13 | 2.00 ±1.00 | 0.00 ±0.00 |
| Male 38 | 35.84 | 2.00 ±1.00 | 0.01 ±0.00 |
| Female 48 | 31.34 | 3.00 ±0.00 | 0.06 ±0.01 |
| Male 50 | 33.64 | 4.00 ±1.00 | 0.05 ±0.01 |
| Male 49 | 36.92 | 4.00 ±1.00 | 0.08 ±0.01 |
| Male 46 | 16.00 | 13.00 ±4.00 | 0.04 ±0.01 |
| Male 31 | 10.24 | 21.00 ±6.00 | 0.05 ±0.02 |
| Female 02 | 16.52 | 0 | 0 |
| Female 03 | 13.97 | 0 | 0 |
| Female 04 | 16.76 | 0 | 0 |
| Female 05 | 23.78 | 0 | 0 |
| Female 08 | 27.70 | 0 | 0 |
| Female 09 | 17.80 | 0 | 0 |
| Female 16 | 22.77 | 0 | 0 |
| Female 17 | 18.41 | 0 | 0 |
| Female 18 | 9.09 | 0 | 0 |
| Female 19 | 10.22 | 0 | 0 |
| Female 20 | 14.43 | 0 | 0 |
| Female 21 | 20.77 | 0 | 0 |
| Female 22 | 10.87 | 0 | 0 |
| Female 23 | 14.87 | 0 | 0 |
| Female 26 | 7.34 | 0 | 0 |
| Female 34 | 39.87 | 0 | 0 |
| Male 11 | 31.93 | 0 | 0 |
| Male 13 | 21.15 | 0 | 0 |
| Male 14 | 27.54 | 0 | 0 |
| Male 15 | 28.56 | 0 | 0 |
| Male 30 | 20.63 | 0 | 0 |
| Male 32 | 20.66 | 0 | 0 |
| Male 36 | 45.81 | 0 | 0 |

**Table S2**: K-fold cross validation value scores for each season’s top performing model. Average used and available spearman rank coefficients and standard deviation (SD) values come from 100 repetitions.

| Season | Used (x̄ = SD) | Available (x̄ = SD) |
| --- | --- | --- |
| *Winter* | 0.97 (0.00) | 0.01 (0.26) |
| *Spring* | 1.00 (0.00) | 0.03 (0.25) |
| *Summer* | 1.00 (0.00) | 0.02 (0.29) |
| *Fall* | 1.00 (0.00) | -0.04 (0.25) |

**Table S3**: Top Resource Selection Models for 24 female GPS-collared sheep in Banff National Park between 2020-2022.

| **Season** | **Model** | **K** | **AICc** |
| --- | --- | --- | --- |
| *Winter* | OBSERVED ~ Elevation.s + I(Elevation.s^2) + Aspect + Slope.s + Esca_distance.e + Wtr_distance.e + Rugged7 + Snow_depth + Forest + Shrubs + Grass_forbs + Rock_rubble + (1\|id) | 12 | 789601.2 |
|  | OBSERVED ~ Elevation.s + I(Elevation.s^2) + Aspect + Slope.s + Esca_distance.e + Wtr_distance.e + Rugged7 + Snow_depth + Forest + Shrubs + Grass_forbs + Rock_rubble + **Bison_UD** + (1\|id) | 13 | 789316.6 |
| *Spring* | OBSERVED ~ Elevation.s + I(Elevation.s^2) + Aspect + Slope.s + Esca_distance.e + Wtr_distance.e + Rugged7 + Shrubs + Grass_forbs + Rock_rubble + Snow_water + (1\|id) | 11 | 307577.1 |
|  | OBSERVED ~ Elevation.s + I(Elevation.s^2) + Aspect + Slope.s + Esca_distance.e + Wtr_distance.e + Rugged7 + Shrubs + Grass_forbs + Rock_rubble + Snow_water + **Bison_UD** + (1\|id) | 12 | 307480.3 |
| *Summer* | OBSERVED ~ Elevation.s + I(Elevation.s^2) + Aspect + Slope.s + Esca_distance.e + Wtr_distance.e + Rugged7 + NDVI.s + Forest + Shrubs+ Grass_forbs + Rock_rubble + Snow_water | 13 | 416212.2 |
|  | OBSERVED ~ Elevation.s + I(Elevation.s^2) + Aspect + Slope.s + Esca_distance.e + Wtr_distance.e + Rugged7 + NDVI.s + Forest + Shrubs+ Grass_forbs + Rock_rubble + Snow_water + **Bison_UD +** (1\|id) | 14 | 416021.4 |
| *Fall* | OBSERVED ~ Elevation.s + I(Elevation.s^2) + Aspect + Slope.s + Esca_distance.e + Wtr_distance.e + Rugged7 + Forest + Shrubs + Grass_forbs + Rock_rubble + (1\|id) | 11 | 225696.6 |
|  | OBSERVED ~ Elevation.s + I(Elevation.s^2) + Aspect + Slope.s + Esca_distance.e + Wtr_distance.e + Rugged7 + Forest + Shrubs + Grass_forbs + Rock_rubble + **Bison_UD** (1\|id) | 12 | 225640.2 |

**Table S4**: Selection coefficients for resource selection models for 24 female GPS-collared sheep in Banff National Park between 2020-2022.

| **Variable** | **Winter** | **Spring** | **Summer** | **Fall** |
| --- | --- | --- | --- | --- |
| Elevation | -0.2 (0.0) *** | -0.6 (0.0) *** | 0.5 (0.0) *** | -0.6 (0.0) *** |
| Elevation^2^ | -0.2 (0.0) *** | -0.3 (0.0) *** | -0.4 (0.0) *** | -0.4 (0.0) *** |
| Aspect | 0.3 (0.0) *** | 0.5 (0.0) *** | 0.3 (0.0) *** | 0.5 (0.0) *** |
| Slope.s | 0.3 (0.0) *** | 0.2 (0.0) *** | 0.0 (0.0) * | 0.5 (0.0) *** |
| Esca.distance | -0.9 (0.0) *** | -1.5 (0.0) *** | -0.2 (0.0) *** | -0.3 (0.0) *** |
| Wtr.distance | 1.5 (0.0) *** | 2.2 (0.0) *** | 0.8 (0.0) *** | 2.0 (0.0) *** |
| Rugged | 11.6 (0.4) *** | 8.7 (0.5) *** | 0.7 (0.5) | 7.2 (0.8) *** |
| Snow.depth | -2.6 (0.0) *** | - | - | - |
| Forest | 1.8 (0.1) *** | - | -0.8 (0.0) *** | 0.5 (0.1) *** |
| Shrubs | 3.0 (0.1) *** | 1.3 (0.0) *** | -0.1 (0.0) * | 2.3 (0.1) *** |
| Grass.forbs | 3.4 (0.1) *** | 2.2 (0.0) *** | -0.5 (0.0) *** | 2.5 (0.1) *** |
| Rock.rubble | 1.6 (0.1) *** | 0.3 (0.0) *** | -1.0 (0.0) *** | 0.7 (0.1) *** |
| Snow.water | - | -2.5 (1.0) * | -1.6 (0.2) *** | - |
| NDVI | - | - | -0.3 (0.0) *** | - |
| Num.Obs. | 107039 | 42615 | 54585 | 31312 |
| RMSE | 0.71 | 0.70 | 0.71 | 0.70 |

** p < 0.05, ** p < 0.01, *** p < 0.001*

**Table S5**: Summary statistics for 29 interactions for bison and sheep between October 19, 2019, and November 23, 2022, in BNP.

| **Stats** | **Results** |
| --- | --- |
| n Fixes | 254690 |
| n Contacts | 175 |
| n Phases | 29 |
| Longest Phase (secs) | 129590 |
| Mean Phase (secs) | 40631 |
| Median Phase (secs) | 32419 |
| no. one-fix Phases | 6 |

**Table S6**: Summary of our GAM model using a bivariate smoother on time to contact and distance to bison to test for effects of an interaction on sheep movement rates.


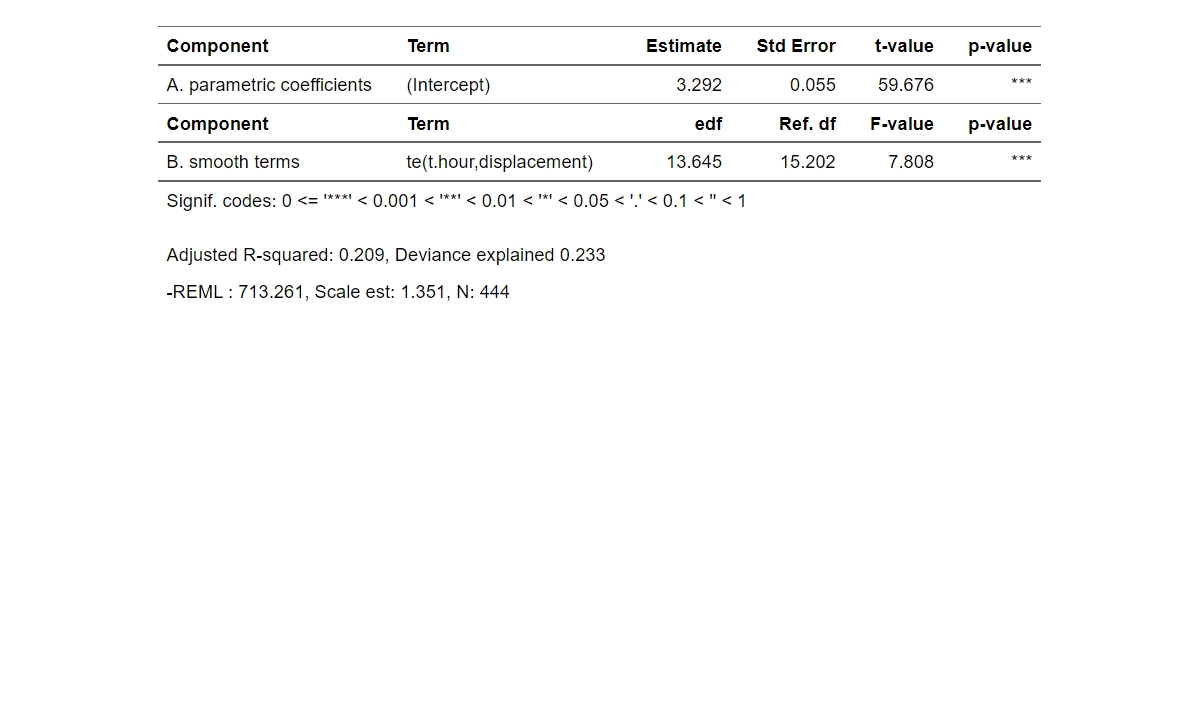

Supplement: Supplementary file 1 — Tables S1–S6. [file ECE3-14-e11008-s001.docx]
